# Choosing a Second Biologic Agent for Inflammatory Bowel Disease Patients—Cost Considerations and Clinical Outcomes

**DOI:** 10.1093/crocol/otaa032

**Published:** 2020-04-29

**Authors:** Kofi Clarke

**Affiliations:** Penn State College of Medicine, Hershey, Pennsylvania, USA

The rising direct health care costs over the past 10 years attributable to Inflammatory Bowel Disease (IBD) is largely driven by expenditures on biological medications.^1^ The spectrum of medication options with varied mechanisms of action for the treatment of IBD has increased in recent years. These newer options and various anti-tumor necrosis factor (anti-TNF) therapies are used as either first-line therapy or as an option when therapy is switched.

With an appropriate emphasis on value-based health care, cost implications of managing complex IBD patients are becoming increasingly important. However, *non-medical switching* or discontinuation of anti-TNF therapies is associated with worse clinical outcomes and increased health care resource utilization.^2^ In addition, uncontrolled moderate to severe IBD leads to a negative impact on the quality of life, work productivity, and increased risk for IBD-related surgeries and hospitalizations.^3^

Measuring validated clinical outcomes or surrogate markers of clinical outcomes whiles on different biologic therapies with different costs provides some guidance for cost-effective management in IBD.

In this issue of *CC360*, Chiorean et al report their findings on healthcare resource utilization and associated costs in a cohort of IBD patients switching to a second biologic agent. Their retrospective study titled “Economic Outcomes of Inflammatory Bowel Disease Patients Switching to a Second Anti-Tumor Necrosis Factor or Vedolizumab” lends some insight to costs implications versus outcomes of switching out of class in patients failing initial anti-TNF agents.

The total and treatment costs were generally higher for patients switching to vedolizumab. The exception was in patients with ulcerative colitis who switched to golimumab. Regarding outcomes, adalimumab and other switchers had *fewer all-cause and IBD-related outpatient visits*.

Our treatment choices when switching biologics in IBD patients should primarily be guided by each unique clinical scenario. This is particularly pertinent when there is not always a benefit in outcomes from using newer therapies

The cohort studied was derived from claims data and disease severity was determined by proxy variables. In addition, there are other factors contributing to the costs including point of care costs, and unique contracts between health care systems and payers which cannot be evaluated in this study design.

However, this study is one more reminder to be cognizant of economic implications versus desirable outcomes when switching biologic therapy.
